# Trapping photons with optical black hole

**DOI:** 10.1038/s41377-023-01122-5

**Published:** 2023-03-14

**Authors:** You-Ling Chen, Qi-Tao Cao, Yun-Feng Xiao

**Affiliations:** 1 State Key Laboratory on Integrated Optoelectronics, Institute of Semiconductors, Chinese Academy of Sciences, Beijing, 100083 China; 2 Frontiers Science Center for Nano-optoelectronics & School of Physics, Peking University, Beijing, 100871 China; 3 Collaborative Innovation Center of Extreme Optics, Shanxi University, Taiyuan, 030006 Shanxi China

**Keywords:** Optics and photonics, Physics

## Abstract

An optical black-hole cavity based on transformation optics enables Q-factor enhancement and strong field confinement, by eliminating the intrinsic radiation loss of the conventional whispering-gallery modes, holding potential for applications in energy harvesting and optoelectronics.

## Introduction

The interaction between light and matter plays a key role for both fundamental research and advanced applications. Whispering-gallery-mode (WGM) cavities, featuring high *Q* factors and small mode volumes *V*, provide a promising platform for intensively enhancing light-matter interaction, which lays the foundation for the realms of ultralow threshold lasers, ultra-sensitive sensing, nonlinear optics and quantum information processing^1,2^. In order to further improve the interaction enhancement, an intuitive way is to compress the mode volumes, which, however, leads to the increasing radiation loss, especially for a small cavity whose geometrical size is comparable to the resonant wavelength. Generally, the radiation loss is resulted from the light tunneling through the curved boundary^1,3^. Over the past few years, tremendous efforts have been paid to compensate for the radiation loss in small WGM cavities for stronger light-matter interaction^4,5^, including surface plasmonic resonance and anisotropic dielectric resonators. Nevertheless, the former would also introduce extra Ohmic loss from the metallic material, and the implementation of the latter is difficult for natural materials.

**Fig. 1:**
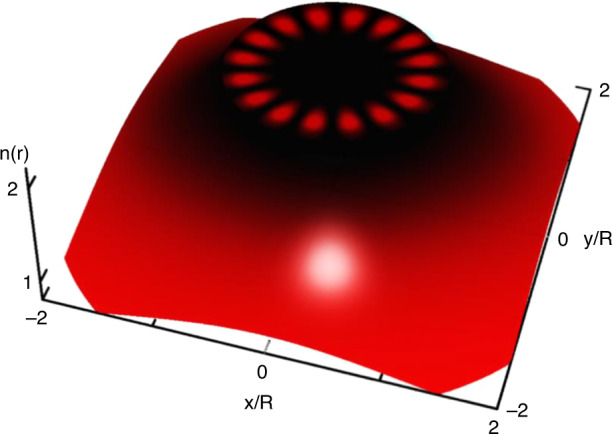
Schematic illustration of a whispering-gallery mode in an optical black-hole cavity

Transformation optics offers a method to manipulate the propagation of light rays and the distribution of electromagnetic fields, enabling light confinement and elimination of intrinsic radiation loss. In a recent publication in this issue of eLight, a team of scientists from Xiamen University in China has proposed and experimentally demonstrated a high-Q optical black-hole (OBH) cavity consisted of a gradient-index cladding structure (Fig. 1), of which both the Q-factor enhancement and tightly confined WGM fields were obtained in the microwave spectra^6^.

By extending the concept of black holes into the optical microcavity, the novel light bending and trapping phenomena by OBHs have been studied, which can also mimic the cosmology effects predicted by general relativity^7–9^. The researchers design a class of OBH cavities based on the conformal mapping of the planar dielectric media by re-examine the total internal reflection effect (TIR) in straight space. The WGM field is revealed with the OBH cladding, following an unconventional 1/r^α^ decay rule, in contrast to the traditional form of the Hankel function in a homogeneous cavity. Theoretically, it is found that the radiation loss can be eliminated perfectly in an ideal OBH cavity, thanks to the infinitely-wide optical potential barrier of OBH cladding. Experimentally, they demonstrate a truncated OBH cavity with a Q-factor enhancement of ~8 and improved field confinement in comparison with a homogeneous cavity. The strategy is further expanded to cavities with asymmetric shapes, and significant Q factor enhancement of 2 orders of magnitude is achieved for a face shaped cavity.

The presented work overcomes the long-lasting fundamental incompatibility between the higher Q-factor and smaller mode volumes in cavities at wavelength scale with arbitrary boundary shapes. More importantly, the technique of designing and customizing the surface field distribution of cavities via transformation optics sheds light on a wide range of fundamental research and potential photonic applications, such as the chaotic dynamics in deformed cavities^10,11^, and non-Hermitian physics in multi-core cavities with gain and loss^12,13^. The OBH cavity would be also expected at the optical frequency band, which may rely on the gradient-thickness waveguide structures^9^ or gradient-size nanostructures^14^. Further efforts will still be required to realize the OBH cavity in higher dimensions for tailoring the interaction between light and matter.
